# Supplementary material for: Structural basis of denuded glycan recognition by SPOR domains in bacterial cell division
Source: Nat Commun. 2019 Dec 5;10:5567. doi: 10.1038/s41467-019-13354-4 (PMC6895207; doi:10.1038/s41467-019-13354-4)
Supplement: Supplementary file 1 — Supplementary Information [file 41467_2019_13354_MOESM1_ESM.pdf]

Supplementary Information

Structural basis of denuded glycan recognition by SPOR domains in bacterial cell division

Martín Alcorlo[a], David A. Dik[b], Stefania De Benedetti[b], Kiran V. Mahasenan[b], Mijoon Lee[b], Teresa Domínguez-Gil[a], Dusan Hesek[b], Elena Lastochkin[b], Daniel López[c], Bill Boggess[b], Shahriar Mobashery[b,1] Juan A. Hermoso[a,1]

[a]Department of Crystallography and Structural Biology, Instituto de Química-Física "Rocasolano", Consejo Superior de Investigaciones Científicas, Madrid, Spain; [b]Department of Chemistry and Biochemistry, University of Notre Dame, Notre Dame, Indiana 46556, United States. [c]National Centre for Biotechnology, Spanish National Research Council (CNB-CSIC), 28049 Madrid, Spain.

[1]To whom correspondence should be addressed. Email: xjuan@iqfr.csic.es; mobashery@nd.edu

Supplementary Table 1. List of primers used for cloning and mutagenesis. Restriction enzyme sites are in bold. Mutated codons are underlined.

	Construct	Primer	Sequence
1	wt	fwd	5′-GCGCGC**CATATG**GCCGATGGCCTGTATCTCCA-3′
		rev	5′-ATATA**CTCGAG**TCAGTCGGGGCGTACCAGCGTCGG-3′
2	R302A	fwd	5′-TCGTGGCGAACCAGCAGAT-3′
		rev	5′-CCGAACTGATAAACACCGG-3′
3	R309A	fwd	5′-TGCACGCGGTGCGGCTGG-3′
		rev	5′-GAATCTGCTGGTTGCGCACG-3′
4	R311A	fwd	5′-GGTGGCCCTGGGACCGA-3′
		rev	5′-CGGTGCAGAATCTGCTGG3′
5	R302A_R309A_R311A	fwd	5′-TGCACGCGGTGGCCCTGG-3′
		rev	5′-GAATCTGCTGGTTCGCCACG-3′
6	Q270A	fwd	5′- ATCTCGCGGTGGGCGCCT-3′
		rev	5′- ACAGGCCATCGGCCATATG-3′
7	F274A	fwd	5′- GCGCCGCCGCCAACCCGG-3′
		rev	5′- CCACCTGGAGATACAGGCC-3′

Supplementary Table 2. Crystallographic data collection and refinement statistics*

	SPOR-RlpA	SPOR-RlpA:1 (soaking)	SPOR-RlpA:1 (Co-crystallization)	SPOR-RlpA:3
**Data collection**				
Wavelength (Å)	0.99999	0.97925	0.97925	0.97918
Space group	$C222_1$	$C222_1$	$C222_1$	$C222_1$
Unit cell *a, b, c* (Å)	67.58, 68.76, 38.77	67.43, 68.15, 38.99	67.56, 68.21, 39.09	67.13, 68.42, 38.86
Unit cell α,β,γ (°)	90, 90, 90	90, 90, 90	90, 90, 90	90, 90, 90
T (K)	100	100	100	100
X-ray source	Synchrotron	Synchrotron	Synchrotron	Synchrotron
Resolution range (Å)	34.38–(1.26–1.21)	47.93–(1.53–1.48)	48.01–(1.35–1.30)	47.92–(1.45–1.40)
Unique reflections	24714 (911)	15044 (754)	22553 (1071)	18010 (923)
Completeness (%)	89.11 (20.20)	98.50 (98.70)	99.70 (99.00)	99.88 (100.00)
Multiplicity	3.0 (1.2)	7.6 (7.6)	12.1 (12.2)	8.8 (9.1)
$R_{merge}{}^{a}$	0.040 (0.186)	0.054 (0.854)	0.038 (1.156)	0.051 (0.884)
$R_{pim}{}^{b}$	0.025 (0.186)	0.020 (0.319)	0.011 (0.340)	0.018 (0.308)
$<I/\sigma(I)>$	16.5 (2.9)	14.8 (1.8)	23.3 (2.1)	20.7 (2.7)
CC1/2	0.99 (0.97)	1.00 (0.80)	1.00 (0.82)	1.00 (0.85)
**Refinement**				
Resolution range (Å)	34.38–1.21	47.93–1.48	48.01–1.30	47.92–1.60
$R_{work}/R_{free}{}^{c}$	0.1529/ 0.1711	0.1689/ 0.1987	0.1685/ 0.1906	0.1564/ 0.1840
No. Atoms				
Protein	586	596	605	627
Water	141	73	84	85
Ligand	–	68	68	66
**R.m.s. deviations**				
Bond length (Å)	0.005	0.006	0.006	0.006
Bond angles (°)	0.78	0.87	0.88	0.86
**Ramachandran** Favored/outliers (%)	100/0	100/0	100/0	100/0
Monomers per AU	1	1	1	1
**Average B-factor**	20.68	29.30	30.33	27.74
Macromolecules	16.81	25.43	26.33	22.46
Ligands	-	52.11	53.16	63.56
Solvent	36.79	39.59	40.64	38.87
**PDB code**	6I05	6I09	6I0A	6I0N

*Values between parentheses correspond to the highest resolution shells

[a]$R_{merge} = \Sigma_{hkl} \Sigma_i \mid I_i(hkl) - [I(hkl)] \mid / \Sigma_{hkl}\Sigma_i I_i(hkl)$, where $\Sigma_i I_i(hkl)$ is the *i*-th measurement of reflection hkl, [I(hkl)] is the weighted mean of all measurements.

[b]$R_{pim} = \Sigma_{hkl}[1/(N-1)]^{1/2} \Sigma_i \mid I_i(hkl) - [I(hkl)] \mid / \Sigma_{hkl}\Sigma_i I_i(hkl)$, where $\Sigma_i I_i(hkl)$ is the *i*-th measurement of reflection hkl, [I(hkl)] is the weighted mean of all measurements and N is the redundancy for the hkl reflection.

[c]$R_{work}/R_{free} = \Sigma_{hkl} \mid F_o - F_c \mid / \Sigma_{hkl} \mid F_o \mid$, where F_c is the calculated and F_o is the observed structure factor amplitude of reflection hkl for the working / free (5%) set, respectively.

Supplementary Table 3. Residues with counterparts in RlpA involved in PG binding among different SPOR domains. Residues contributing with polar interactions through backbone atoms have been omitted.

**RlpA**	**CwlC**	**FtsN**	**DamX**
Gln270	Gln13	Gln251[*1]	Gln351[*2]
Phe274	Phe17	Phe255	
Leu307	Leu45	Trp283[*1]	Trp385[*2]
	Ala192	Ser254[*1]	Ser354[*2]
		Arg256[‡1]	
		I313[*1]	
Basic patch<br>Arg302<br>Arg309<br>Arg331	Lys47<br>Lys42	Arg285[*1]	Arg380<br>Lys418
Additional residues	Asn22→NAM(3)	Asn281[†1]→NAM(1)<br>Gln260→NAM(4)	Trp416[*2]→NAM(1)<br>Trp364[*2]→NAG(4)<br>Asn360[*2]→NAM(3)<br>Asn360[†2]<br>Trp364[†2]<br>Trp385[†2]

*Important residues for PG binding tested *in vivo* and associated reference.

† *In vivo* tested residues (and associated reference) that were shown not to be important for PG binding.

‡ *In vivo* tested residues (and associated reference) that were shown to have a mild effect in PG binding.

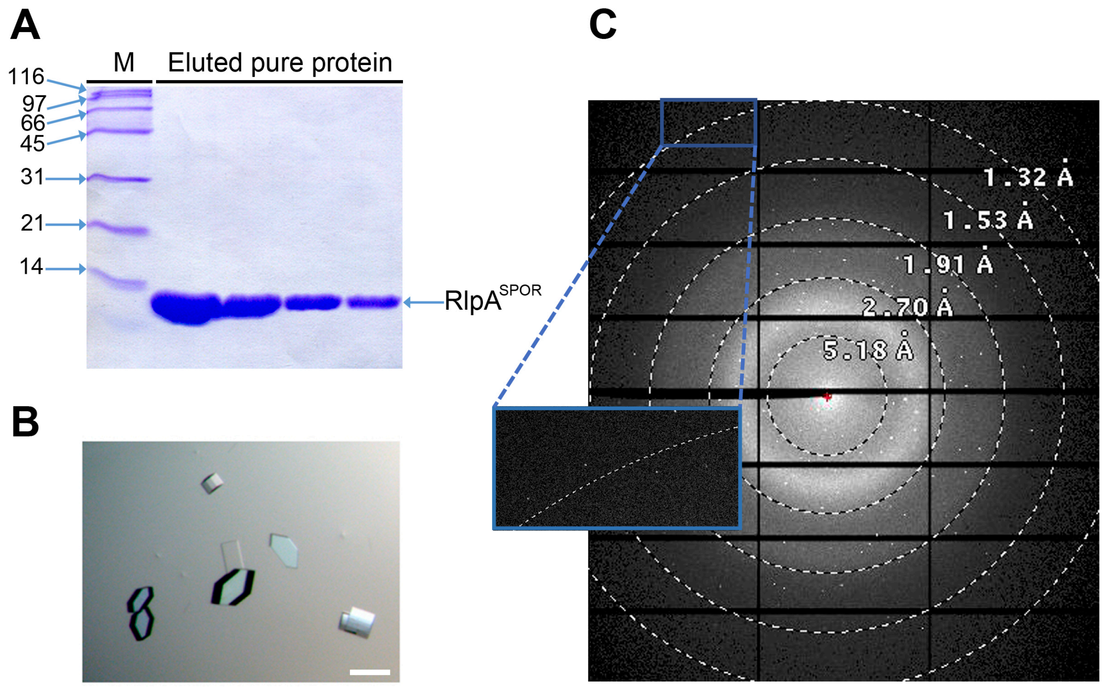

Supplementary Figure 1. SPOR-RlpA purification, crystallization and data collection. (**A**) SDS-PAGE (15%) analysis of the eluted pure SPOR-RlpA after His-tag removal with TEV protease. Weight-molecular markers (kDa) are indicated for the left lane. (**B**) SPOR-RlpA crystals obtained in 0.15 M NaF and 16% PEG 3350 (w/v). Scale bar represents 0.3 mm. (**C**) Diffraction image of SPOR-RlpA crystal collected in beamline XALOC at the ALBA synchrotron using a Pilatus 6M detector. Resolution rings are indicated with dashed lines. The close-up view shows an example of reflections around 1.32 Å resolution and beyond.

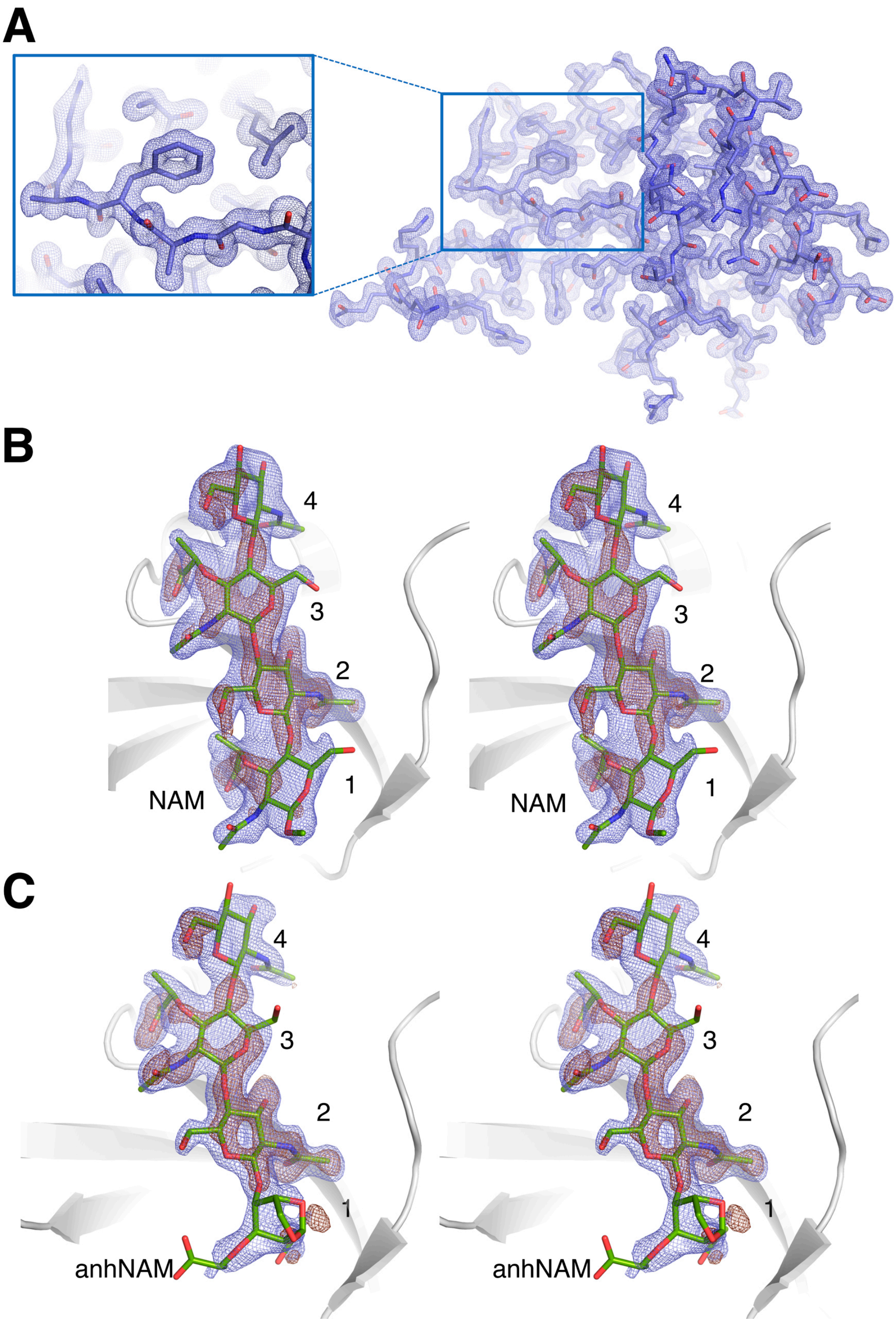

Supplementary Figure 2. Electron density for SPOR-RlpA and compounds 1 and 3. **(A)** Electron-density map (2Fo-Fc map contoured at 1σ) for the 1.2 Å resolution structure of SPOR-RlpA from *P. aeruginosa*. The boxed region shows a close-up view of the map in which electron densities at 1.2 Å resolution can be appreciated. **(B)** Stereoview showing the electron-density map (2Fo-Fc map contoured at 1σ) for compound **1** (in blue) as observed in the co-crystallization SPOR-RlpA:**1** complex at 1.3 Å resolution. Initial Fo-Fc map contoured at 2.5σ is shown superimposed and colored in brown. **(C)** Stereoview showing the electron-density map (2Fo-Fc map contoured at 0.8 σ) for compound **3** (in blue) as observed in the soaking SPOR-RlpA:**3** complex at 1.4 Å resolution. Initial Fo-Fc map contoured at 2.5σ is shown superimposed and colored in brown. The position of the sugar units (depicted in sticks) is indicated with numbers.

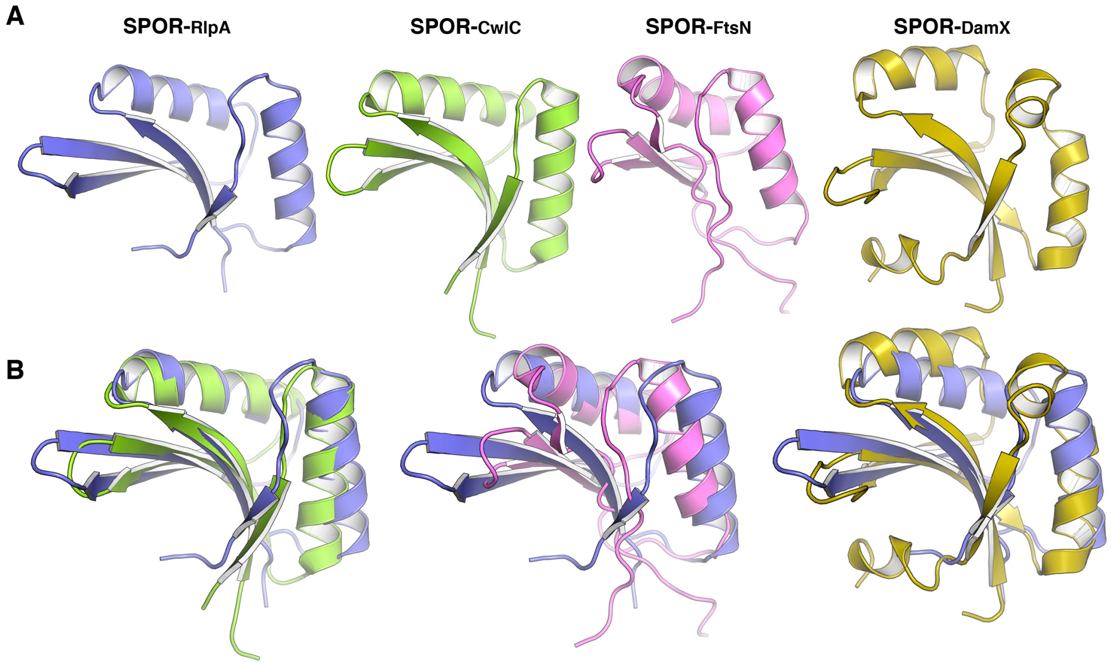

Supplementary Figure 3. Available three-dimensional structures of SPOR domains (**A**) Three-dimensional structures of SPOR-RlpA from *P. aeruginosa* (this work, blue ribbon) and all of the SPOR structures available to date. The core fold is an antiparallel β-sheet, flanked on the convex side by two α-helices (green). Notably, SPOR-DamX has an additional helix that is relatively mobile. (**B**) Structural superimposition between SPOR domains from CwlC from *B. subtillis* (green ribbon, PDB 1X60, RMSD= 2.1 Å), FtsN from *E. coli* (pink ribbon, PDB 1UTA, RMSD=2.92 Å), and DamX from *E. coli* (yellow ribbon, PDB 2LFV, RMSD=3.29 Å) with SPOR-RlpA (blue ribbon).

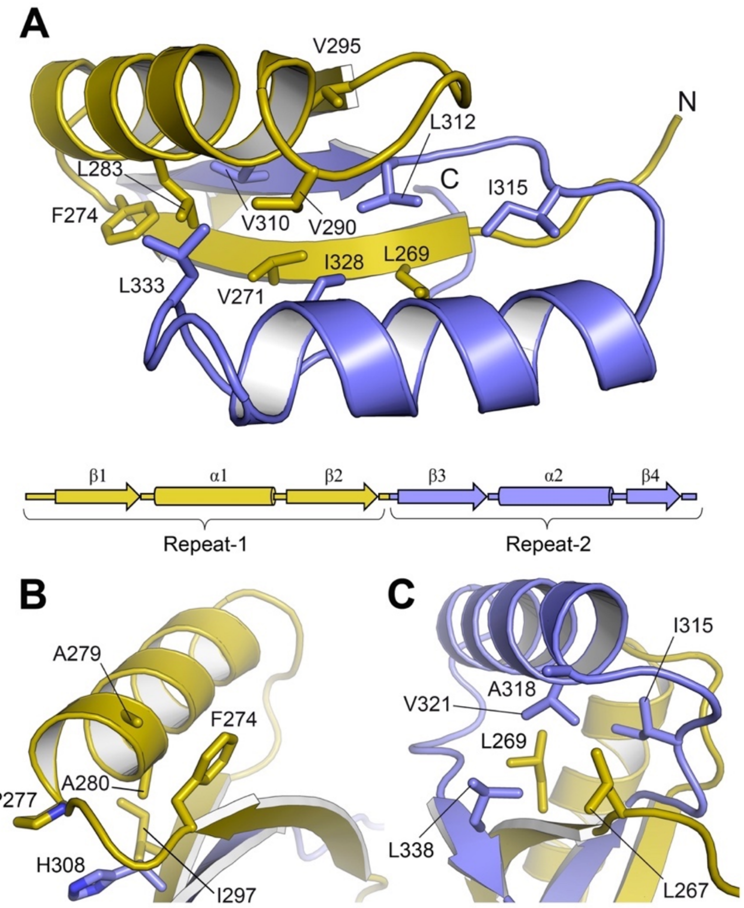

Supplementary Figure 4. Hydrophobic interactions in the core of the SPOR-RlpA. (**A**) Cartoon representation displaying the side chains at the protein core in capped sticks. Repeats 1 and 2 are colored yellow and purple, respectively. The secondary-structure elements of SPOR-RlpA are represented schematically below the structure. The inward-facing residues on the β-sheet and α-helices form a hydrophobic core (L269, V271, F274, L283, V290, V295, V310, L312, and I328) that is expanded at both sites of α2 by the inclusion of I315 and L333, both located in turn regions. (**B** and **C**) Hydrophobic interactions on the edges of the protein stabilize the N-terminal tips of the α-helices on the β-sheet. These contribute to the interaction between the α-helices and the β-sheet. In α1, A279 and A280 interact with F274 from β1 and I297 from β2. P277 interacts with H308 from β3. In α2, V321 interacts with L269 from β1, L338 from β4 and I315 from the turn region. A318 interacts with L267 from β1.

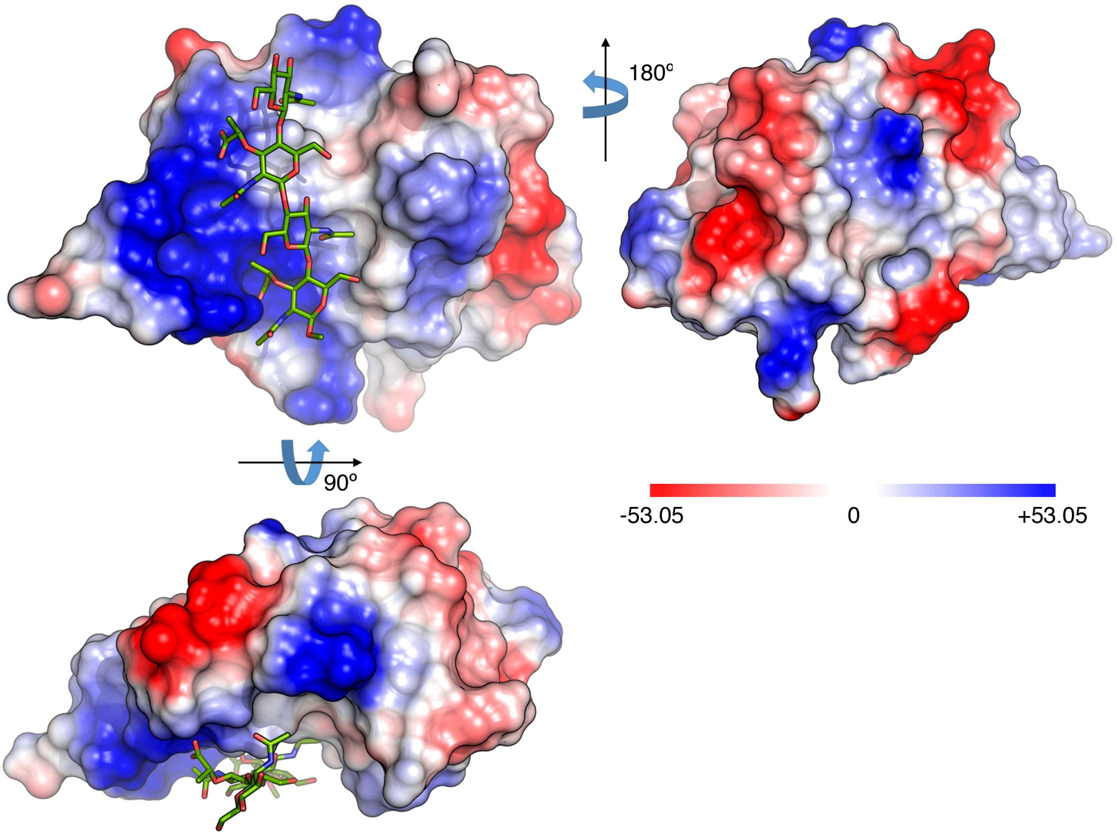

Supplementary Figure 5. Electrostatic potential surface of the SPOR-RlpA domain in complex with compound 1. Compound **1** is represented as capped sticks with C atoms in green. The peptidoglycan-binding site is located only in the basic patch of the concave face of the domain. The color key shows the Poisson-Boltzmann electrostatic-potential surface (color bar range ±53.05 kT/e).

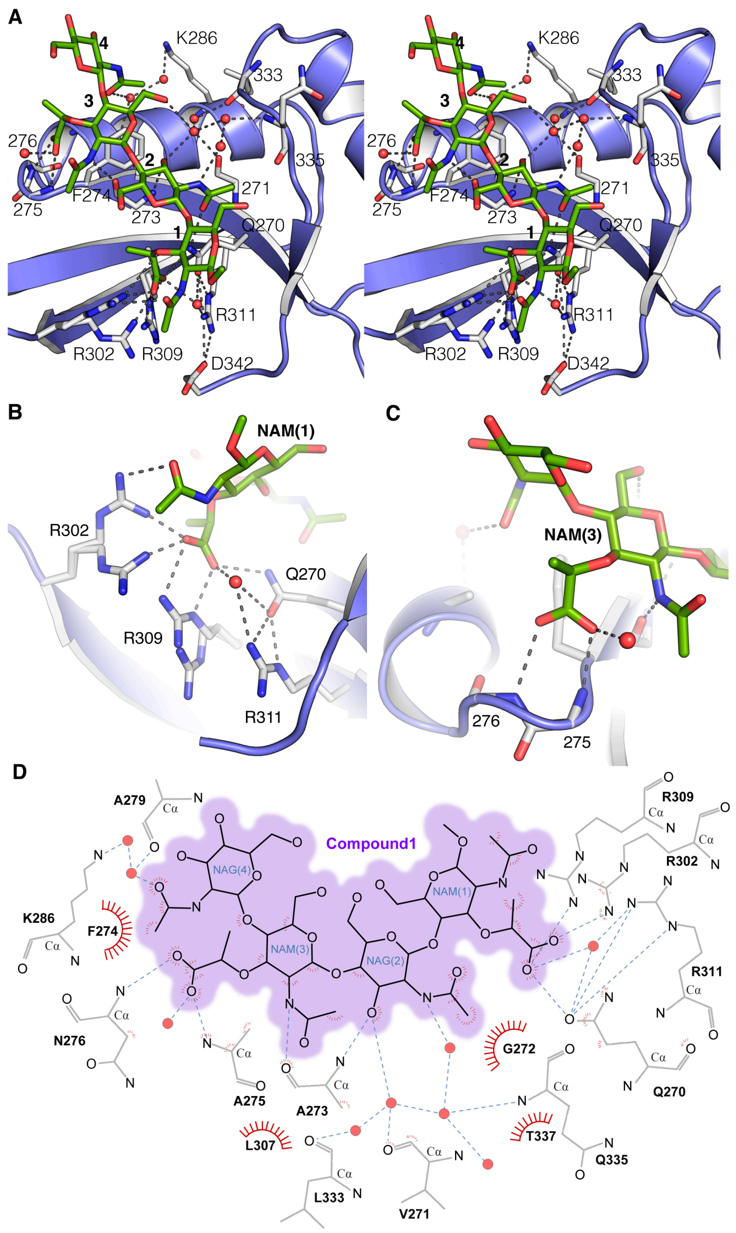

Supplementary Figure 6. Denuded-glycan recognition by SPOR-RlpA. (**A**) Stereoview showing the interactions between denuded PG (compound **1**) and the SPOR domain. Residues involved in PG recognition are labeled, those interacting with PG only through main-chain interactions given by a number. Polar interactions are represented as dotted lines. (**B-C**) Detailed view of the interactions stabilizing the NAM rings at positions 1 and 3. (**D**) Cartoon representation of the interaction network shown in **A**. The cartoon shows hydrogen bonds and hydrophobic interactions represented by dashed blue lines and red hemi-circles with radiating spokes, respectively. Covalent bonds in **1** (center) are in black, protein side chains and main chain atoms are in gray. NAG: *N*–Acetyl glucosamine, NAM; *N*–Acetyl muramic acid (the positions are indicated in parenthesis). Water molecules are shown as red spheres . Atomic interactions were determined using LigPlot [3] with default parameters: for hydrogen-bond calculation parameters maximum H-A distance was 2.70 Å and maximum D-A distance was 3.35 Å, where H=hydrogen, A=acceptor and D=donor. For non-bonded contact parameters minimum contact distance was 2.90 Å a maximum contact distance was 3.90 Å with the option “Hydrophobic-any contacts” activated.

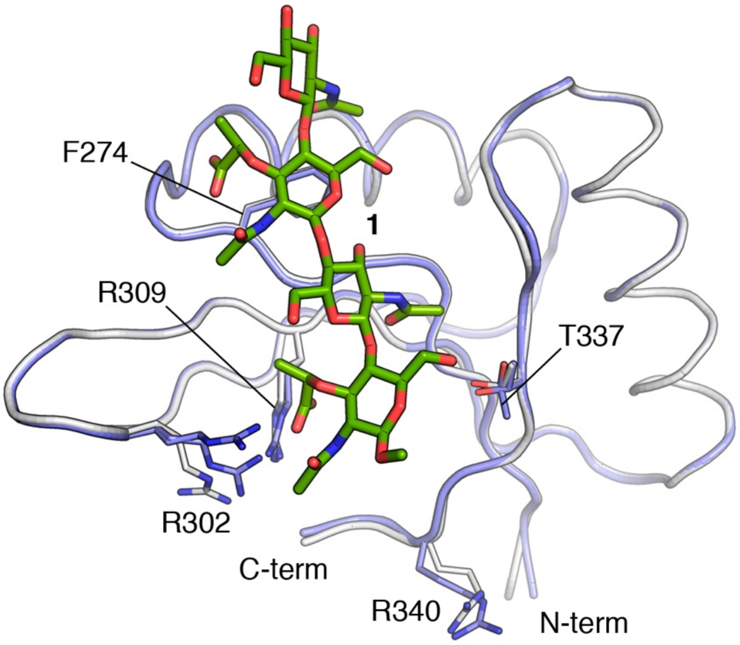

Supplementary Figure 7. Structural comparison between the apo and holo SPOR-RlpA forms. The superimposition shows the structure of the SPOR-RlpA alone (gray) and in complex with compound **1** (blue). Both structures (depicted in a ribbon representation) are very similar with a RMSD value of 0.109 for Cα atoms. Compound **1** is shown with green-capped sticks for carbon atoms. Amino acid residues for which the side chain exhibits distinct orientations in the two structures are shown in sticks. R302 and R309 present two conformations in the presence of compound **1**.

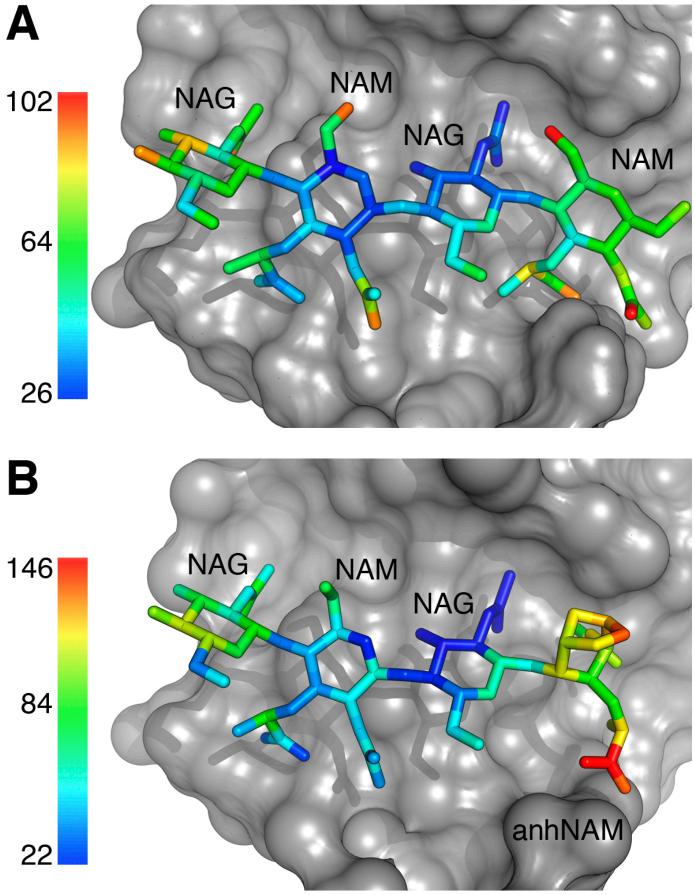

Supplementary Figure 8. Atomic B factors for compounds 1 and 3 as observed in the SPOR-RlpA:1 and SPOR-RlpA:3 complexes. (**A**) Crystal structure of the SPOR domain (gray surface) in complex with compound **1**. (**B**) Crystal structure of the SPOR domain (gray surface) in complex with compound **3**. Ligands are represented as capped sticks and colored according to the B factor distribution, ranging from low (blue) to high (red) values. In both cases, the more stable rings are those at positions 2 and 3. The anhNAM ring in compound **3** presents the highest mobility (panel B).

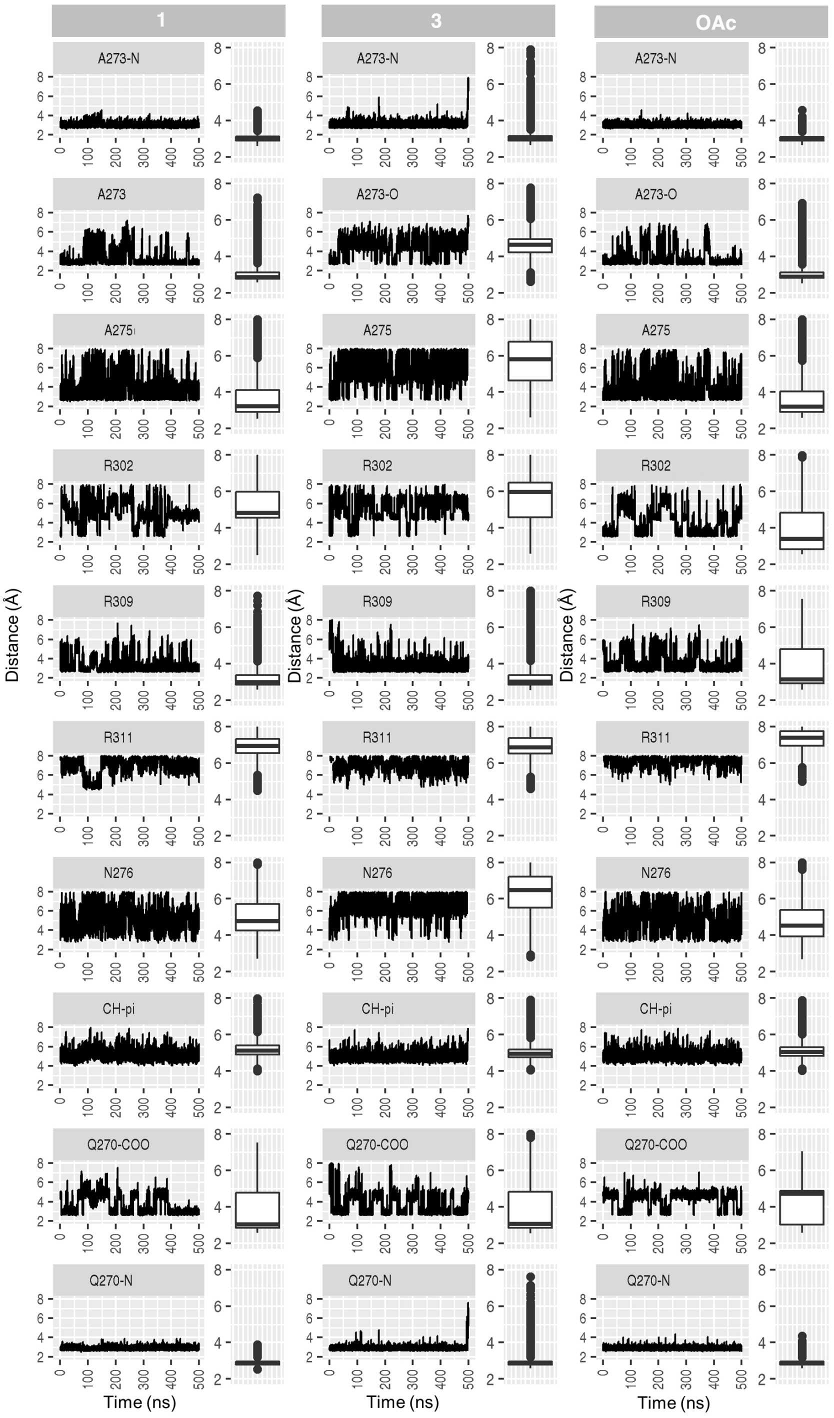

Supplementary Figure 9. **Interactions of the ligands with SPOR-RlpA as a function of time, as calculated during molecular-dynamics simulations.** The variations are depicted as fluctuations in distances (Å) for ten-thousand sampled conformations of protein-ligand complex (complex with compound **1**, compound **3**, and *O*-acetylated PG (**OAc**)) during the 500 ns of MD simulations. The boxes in the middle of the box-plots show the central 50% of the distances, while the lines represent 25% of upper and lower distances. The lines inside the box represent the median value. The dots at the end of lines are outliers. For consistency in plotting the y-axis, the values in the range of 2-8 Å are plotted in the figure for all of the distances. The definition of distances is similar to the SPOR:**1** complex in Figure 8.

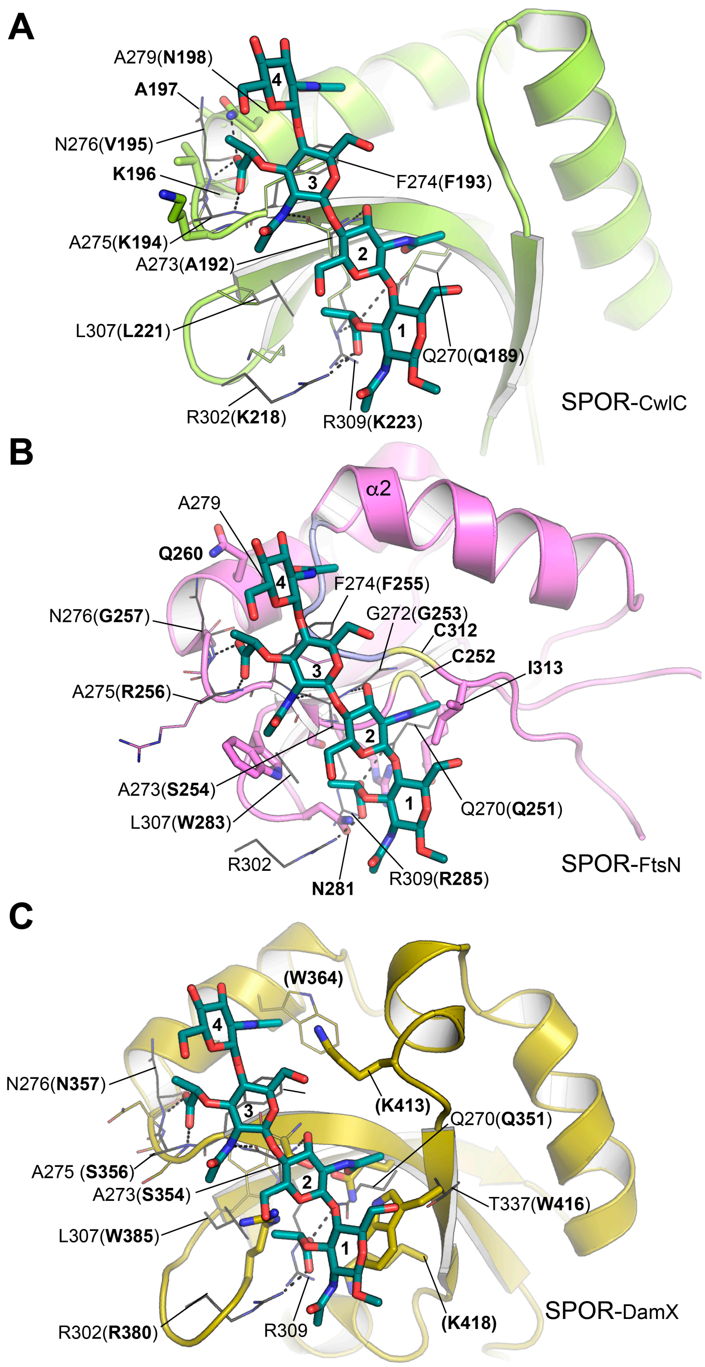

Supplementary Figure 10. Structural comparison between SPOR-RlpA PG-binding determinants and the available SPOR structures. Proposed model of PG (compound **1**) interaction for previously reported SPOR domain structures based on the crystal structure of SPOR-RlpA:**1** complex (this work). Compound **1** is depicted in cyan capped sticks. In all cases, the SPOR-RlpA residues involved in glycan-chain binding are shown as gray lines and labeled. Residues in bold font and in parentheses represent the equivalent residues in each SPOR structure. Hydrogen bonds are shown as dashed lines. (**A**) Solution structure of SPOR-CwlC (PDB code 1X60, in green). The proposed interaction model preserves most of the observed interactions in SPOR-RlpA and explains the predicted role of K194 and N198 (shown in thick sticks) in PG binding [4]. (**B**) Solution structure of SPOR-FtsN (PDB code 1UTA, in pink) with **1** superimposed. While the proposed interaction model preserves most of the observed interactions in SPOR-RlpA:**1**

complex, the binding site is partially occluded by loop connecting α2 with β4 (residues 308 to 313, colored in purple). Interestingly, SPOR-FtsN presents Cys residues (C252 and C312, colored in yellow) in this region, that could rearrange upon disulfide bond formation; nicely explaining the observed experimental correlation between PG binding and disulfide bond formation in FtsN[1]. Reduced septal localization of FtsN upon mutations of residues Q251, S254, W283, R285, and I313 (shown in thick sticks) [1] is also nicely explained in the proposed model. Based on our comparative study, we also propose that Q260 and N281 could be involved in the stabilization of positions 4 and 1 of the ligand, respectively. (**C**) Solution structure of SPOR-DamX (PDB code 2LFV, in yellow) with **1** superimposed. According to Williams *et al.* [2] Q351, S354 and W416 (depicted in thick sticks) play a key role in septal localization of SPOR-DamX. While the first two would reproduce observed interactions in SPOR-RlpA, the W416 (not conserved in RlpA) is nicely placed for stabilizing the NAM(1) ring. Based on our comparative study, we also propose that R380 could be involved in the stabilization of positions 1 or 2 of the ligand, similarly to R302 in SPOR-RlpA. Together with R380, K418 could contribute to the formation of the basic patch. Also, K413, is optimally located to establish polar contacts with ligand positions 2 or 3. Finally, we predict that W364 is involved in NAG(4) stabilization.

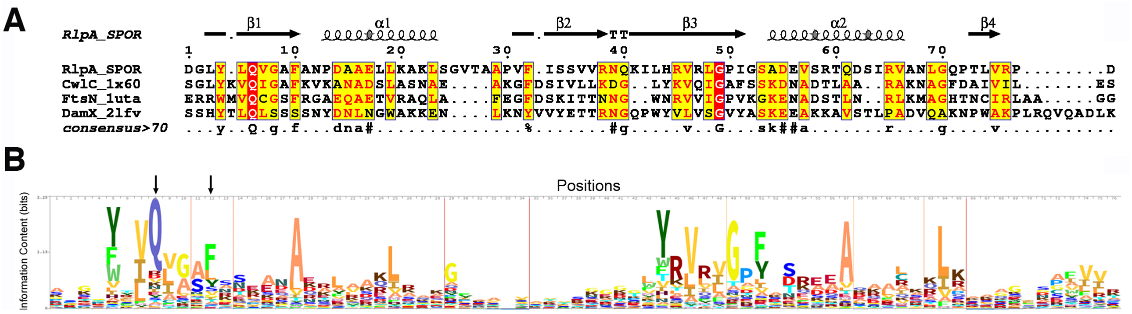

Supplementary Figure 11. Sequence comparison among SPOR domains. (A) Sequence alignment between SPOR-RlpA and the rest of SPOR domains with available three-dimensional structure. Secondary structure elements of SPOR-RlpA are indicated and numbered. Residue numbering follows the SPOR-RlpA sequence. Residues marked with red or yellow boxes indicate sequence identity or similarity, respectively. Sequence alignment was performed with T-Coffee (http://tcoffee.crg.cat/) and the output was processed with EPPript 3.0 (http://espript.ibcp.fr) [5]. (**B**) The HMM (Hidden Markov Model) logo for SPOR domain as calculated by Pfam Database (PF00656, Pfam 31.0) with the 12745 sequences available (December 2018) for SPOR domains. Relative height of letter is proportional to its emission probability. The positions 8 and 12 of the SPOR sequence, mentioned in the main text, are indicated by arrows.

Supplementary References:

1 Duncan, T. R., Yahashiri, A., Arends, S. J., Popham, D. L. & Weiss, D. S. Identification of SPOR domain amino acids important for septal localization, peptidoglycan binding, and a disulfide bond in the cell division protein FtsN. *J Bacteriol* **195**, 5308-5315, doi:JB.00911-13 [pii]
10.1128/JB.00911-13 (2013).
2 Williams, K. B. *et al*. Nuclear magnetic resonance solution structure of the peptidoglycan-binding SPOR domain from Escherichia coli DamX: insights into septal localization. *Biochemistry* **52**, 627-639, doi:10.1021/bi301609e (2013).
3 Laskowski, R. A. & Swindells, M. B. LigPlot+: multiple ligand-protein interaction diagrams for drug discovery. *J Chem Inf Model* **51**, 2778-2786, doi:10.1021/ci200227u (2011).
4 Mishima, M. *et al*. Solution structure of the peptidoglycan binding domain of Bacillus subtilis cell wall lytic enzyme CwlC: characterization of the sporulation-related repeats by NMR. *Biochemistry* **44**, 10153-10163, doi:10.1021/bi050624n (2005).
5 Robert, X. & Gouet, P. Deciphering key features in protein structures with the new ENDscript server. *Nucleic Acids Res* **42**, W320-324, doi:10.1093/nar/gku316 (2014).
