# Supplementary material for: Structural basis of denuded glycan recognition by SPOR domains in bacterial cell division
Source: Nat Commun. 2019 Dec 5;10:5567. doi: 10.1038/s41467-019-13354-4 (PMC6895207; doi:10.1038/s41467-019-13354-4)
Supplement: Supplementary file 3 — Description of Additional Supplementary Files [file 41467_2019_13354_MOESM3_ESM.pdf]

Description of Additional Supplementary Files

File Name: Supplementary Movie 1

Description: Movie showing the 500-ns MD simulation of SPOR-RlpA:1 (X-ray) complex.

File Name: Supplementary Movie 2

Description: Movie showing the 500-ns MD simulation of SPOR-RlpA:3 (X-ray) complex.

File Name: Supplementary Movie 3

Description: Movie showing the 500-ns MD simulation of SPOR-RlpA:OAc (model) complex.
